# Vorstellung eines neuen präemptiven endoskopischen Therapiekonzeptes bei Duodenaleingriffen am Beispiel einer iatrogenen Duodenalperforation nach perkutan-transrenaler Nephrostomie

**DOI:** 10.1007/s00104-025-02256-5

**Published:** 2025-05-22

**Authors:** V. Betz, A. Goerdt, R. Kiesow, J. Müller, B. Riefel, E. Scharsack, U. Zimmermann, M. Reeh, G. Loske

**Affiliations:** Klinik für Allgemein‑, Viszeral‑, Thorax- und Gefäßchirurgie, Kath. Marienkrankenhaus Hamburg gGmbH, Alfredstr. 9, 22087 Hamburg, Deutschland

Anhand eines Fallbeispiels einer iatrogenen Duodenalperforation nach perkutan-transrenaler Nephrostomie wollen wir zeigen, wie ein neues präemptives endoskopisches Therapiekonzept zusätzlich zur operativen Therapie verwendet werden kann.

## Falldarstellung, diagnostisches und operatives Vorgehen

Bei einem 65-jährigen Patienten war aufgrund einer unklaren symptomatischen Ektasie Grad II des Nierenbeckenkelchsystems (NBKS) rechts mit unklarer endoluminaler Raumforderung bei vorbestehendem Prostatakarzinom und einem multiplen Myelom eine perkutan-transrenale Nephrostomie ambulant angelegt worden.

**Abb. 1:**
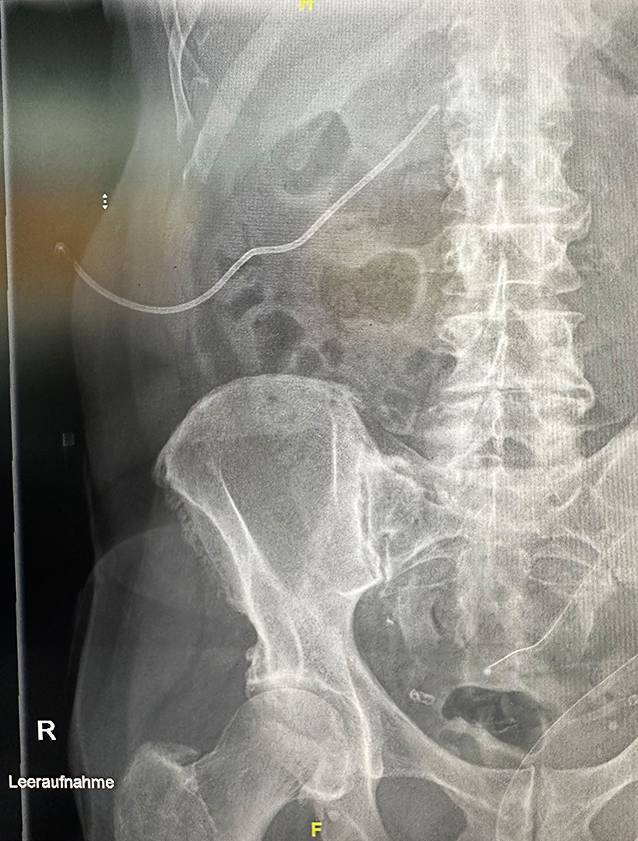
Einliegender Nephrostomiekatheter, Röntgenleeraufnahme zum Aufnahmezeitpunkt

Nach 5 Tagen wurde der Patient aufgrund des klinischen Verdachts auf eine Fehllage des Nephrostomiekatheters notfallmäßig vorstellig. Über den Katheter entleerte sich galliges Sekret. Schmerzen bestanden nicht, auch kein Fieber oder eine Makrohämaturie. Laborchemisch bot sich kein Anstieg der Infektparameter. Sonographisch fand sich das NBKS rechts zweit- bis drittgradig ektatisch, links ungestaut. Nativ-röntgenologisch in einer Ebene zeigte sich die Nephrostomie von rechts mit Ende in Projektion auf das Nierenbecken auf Höhe des rechten Querfortsatzes LWK 1 (Abb. 1).

Bei eindeutiger klinischer Symptomatik einer Katheterfehllage erfolgte die notfallmäßige explorative Laparotomie. Nach Kocher-Mobilisation bestätigte sich, dass die Katheterspitze aus dem unmittelbar nebenliegenden Nierenbecken in das Duodenum gelegt worden war. Der Katheter wurde entfernt und sowohl der Defekt in der Nierenkapsel als auch der Duodenaldefekt mit 4‑0-Polydioxanon-Naht (MonoPlus®, B. Braun SE, Melsungen, Deutschland) verschlossen.

Bei weiterhin stark aufgestauter rechter Niere und erhöhtem Serumkreatininwert wurde durch die urologischen Kollegen unseres Hauses noch am 1. postoperativen Tag eine neuerliche perkutan-transrenale Nephrostomie rechts eingelegt.

Wir gehen davon aus, dass von den meisten Lesern der alleinige operative duodenale Defektverschluss per Naht sowie die üblichen supportiven Therapiemaßnahmen wie Antibiotikagabe und parenterale Flüssigkeitszufuhr als ausreichend angesehen werden.

Nach dem Eingriff würde sich die Verlaufsbeobachtung mit Kontrolle der Infektparameter anschließen und abgewartet werden, ob der klinische Heilungsverlauf ungestört ist, sodass von einem suffizienten Verschluss des Defektes ausgegangen werden kann.

Eingriffe am Duodenum sind häufig initial mit einer Passagestörung vergesellschaftet. Bei indiziertem Notfalleingriff, Vollnarkose und Laparotomie wird daher in der Regel prä-, peri- und postoperativ eine passive gastroduodenale Ableitsonde transnasal platziert. Die Lage der Sonde wird im Rahmen der Operation digital kontrolliert und die Sonde nach dem Eingriff als Überlaufsonde für die erste postoperative Phase belassen. Bei fehlendem Reflux würde die Drainage entfernt werden und der Kostaufbau erfolgen.

### Frage:

Welche zusätzliche endoskopische Behandlungsmethode könnte ergänzend zum alleinigen operativen Defektverschluss des Duodenums genutzt werden?

### Lösung:

Die endoskopische Unterdrucktherapie im Duodenum kann auch als präemptive Maßnahme zur Anastomosenprophylaxe genutzt werden.

Im präemptiven Einsatz der endoskopischen Unterdrucktherapie (EVT) bei Ösophagektomie, die wir in Form einer präemptiven aktiven Refluxdrainage (PARD) einsetzen, haben wir vielfach den positiven Nutzen der aktiven Elimination der galligen Verdauungssekrete auf die Anastomosenheilung gesehen.

Die EVT ist zur Behandlung duodenaler Defekte im postoperativen Komplikationsmanagement sowie bei Perforationen geeignet. Der Schlüssel zum Erfolg der Therapie von Duodenaldefekten liegt neben dem Defektverschluss insbesondere in der effektiven, nach intraluminal gerichteten Drainage der duodenalen Verdauungssekrete. Wenn diese Drainage effektiv gelingt, wird die Wundkontamination gestoppt, und die Defekte können abheilen.

Der präemptive Behandlungsansatz der Nutzung aktiver Unterdruckdrainagen lässt sich auch auf die Anwendung bei Operationen im Duodenum übertragen. Das zugrunde liegende Behandlungsprinzip ist einfach: Statt einer passiven Überlaufdrainage, die nie eine vollständige Sekretableitung bewirkt, lassen sich mit der Unterdrucktherapie aktiv die Verdauungssekrete eliminieren. Statt einer passiven gastroduodenalen Ableitsonde nutzen wir eine mit einer offenporigen Folie ausgerüstete Ableitsonde, an der gesaugt werden kann.

Diesen einfachen Therapieansatz der aktiven luminalen Sekretableitung haben wir als zusätzliche präemptive Behandlungsmaßnahme auch in unserem Fall benutzt, um die Abheilung der Duodenalnaht zu sichern.

Wir stellen die Methode der präemptiven intraluminalen endoskopischen Unterdrucktherapie (PINT) des Duodenums vor und skizzieren den weiteren Behandlungsverlauf.

## Materialien und Methoden

Ziel der präemptiven Methode PINT ist es, die duodenalen Verdauungssekrete aus dem Duodenallumen zu evakuieren und diese in der ersten vulnerablen Phase der Wundheilung von der Duodenalnaht fernzuhalten.

Wenige Stunden nach dem operativen Eingriff initiierten wir zur Prophylaxe einer Duodenalnahtinsuffizienz die PINT des Duodenums mit Abdeckung des Perforationsdefektes und Ableitung des galligen Sekretes mittels einer doppellumigen offenporigen Foliendrainage (dOFD). An das eine Lumen der Sonde kann ein Unterdruck angelegt werden, welcher darüber der Sekretabsaugung dient. Das andere Lumen entspricht einer Ernährungssonde, hierüber können Patienten simultan zur Unterdruckausübung enteral ernährt werden.

**Abb. 2:**
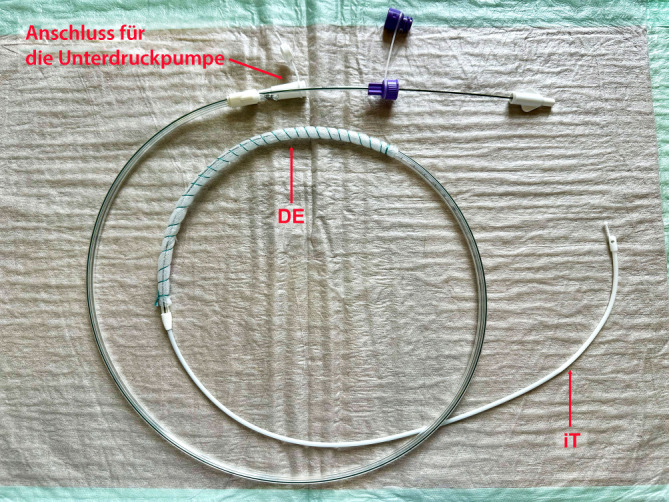
Doppellumige offenporige Foliendrainage (dOFD): Drainageelement (DE) mit folienummanteltem gastralem Schenkel und Ernährungssonde (iT)

Die dOFD wurde mit einer Trelumina Sonde (Freka® Trelumina, Fresenius SE & Co. KGaA, Bad Homburg, Deutschland) und der offenporigen Drainagefolie (Suprasorb® CNP Drainagefolie, Lohmann & Rauscher International GmbH & Co. KG, Rengsdorf, Deutschland) hergestellt. Als Drainageelement wurde der gastrale Ableitschenkel der Sonde über eine Länge von insgesamt 25 cm mit der dünnen Folie ummantelt. Zur Fixierung der Drainagefolie verwendeten wir eine Fadenumwickelung mit Polyesterfaden (Mersilene®, Ethicon Deutschland Johnson & Johnson, Norderstedt, Deutschland). Der Belüftungsschenkel der Sonde wurde mit einer Klemme verschlossen, er wird nicht benötigt (Abb. 2).

**Abb. 3:**
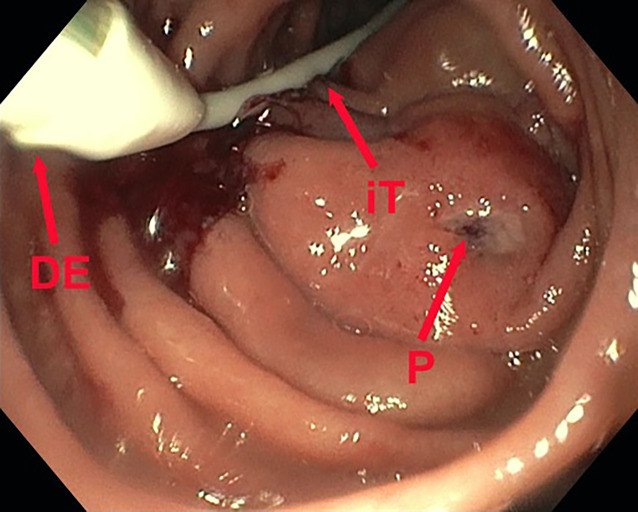
Platzierungsmanöver der dOFD. Ernährungssonde (iT), Übergang zum folienummantelten Abschnitt (DE), übernähter Perforationsdefekt (P) des Nephrostomiekatheters mit perifokalem Ödem und lokalen Entzündungszeichen

Die dOFD hat einen Durchmesser von nur 6 mm. Die transnasale Einführung erfolgte unter endoskopischer Sicht in gleicher Technik wie bei einer gastroduodenalen Ableitsonde. Der intestinale Ernährungsschenkel der dOFD wurde endoskopisch entlang des Pylorus in das Duodenum vorgeschoben und die dOFD mit dem offenporigen Folienabschnitt durch weiteres Vorschieben tief im Duodenum, die Defektregion überdeckend, platziert (Abb. 3).

Ein kontinuierlicher Unterdruck von −125 mm Hg wurde mit einer elektronischen Unterdruckpumpe (Suprasorb® CNP Endo-Therapieeinheit, Lohmann & Rauscher International GmbH & Co. KG, Rengsdorf, Deutschland) an das Drainageelement angelegt.

Durch die Anlage des Unterdrucks werden die duodenalen Sekrete permanent abgesaugt, das Duodenallumen kollabiert und wird dekomprimiert.

Über die weit distal von der Unterdruckausübung einliegende integrierte Ernährungssonde kann simultan zur Unterdruckausübung die intestinale Ernährung erfolgen. Bereits ab dem 1. postoperativen Tag erhielt der Patient Sondenkost, welche rasch auf eine Kalorienzufuhr von 2000 kcal gesteigert werden konnte. Eine supportive parenterale Ernährung war nicht erforderlich. Zur Mundbefeuchtung durfte der Patient schluckweise Wasser trinken, dieses wurde ebenfalls abgesaugt.

Entsprechend unserem Algorithmus für die endoskopische Unterdrucktherapie (ENPT) führten wir eine endoskopische Kontrolle der inneren Wundsituation mit Drainagewechsel der dOFD alle 3 bis 4 Tage durch. Es wurde eine endoskopische Begutachtung der intraluminalen Nahtverhältnisse vorgenommen und entschieden, ob die Therapie beendet oder fortgeführt wird. Für die Endoskopie verwendeten wir CO_2_-Untersuchungsgas.

Die perioperativ begonnene intravenös-antibiotische Therapie wurde fortgeführt.

## Ergebnisse

**Abb. 4:**
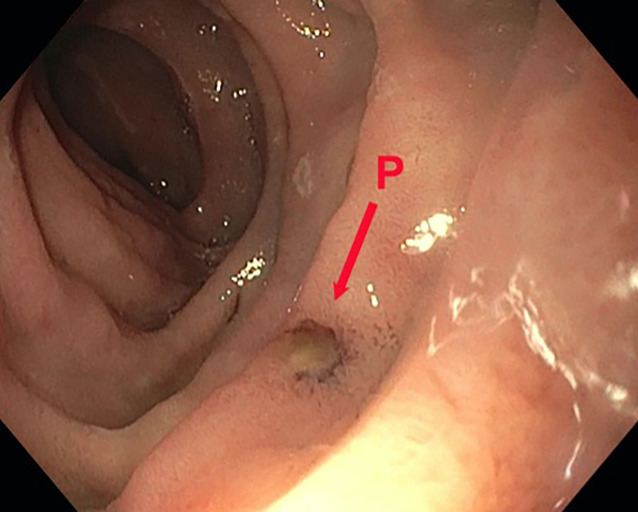
Perforationsdefekt (P) am 4. postoperativen Tag, Wechsel der Drainage mit Inspektion der inneren Wunde. Man sieht keinerlei gallige Imbibierung der Wunde, das gallige Sekret wurde komplett abgesaugt. Die Perforationsstelle stellt sich reizlos mit einem minimalen Fibrinbelag dar, kein Defekt nachweisbar

In der ersten endoskopischen Kontrolle nach 3 Tagen zeigten sich regelhafte, reizlose Nahtverhältnisse mit leichtem Fibrinbelag auf der Naht ohne weitere Entzündungszeichen und ohne Defekt bei guten Durchblutungsverhältnissen. Das initiale perifokale Ödem war rückläufig. Die dOFD war nicht disloziert und lag mit dem Drainageelement im Duodenum. Es wurde eine neue dOFD problemlos endoskopisch platziert und die präemptive Therapie noch für eine zweite Periode fortgeführt (Abb. 4).

Die nachfolgende Kontrolle nach weiteren 3 Tagen bot eine reizlose innere Duodenalwunde ohne lokale Infektzeichen.

**Abb. 5:**
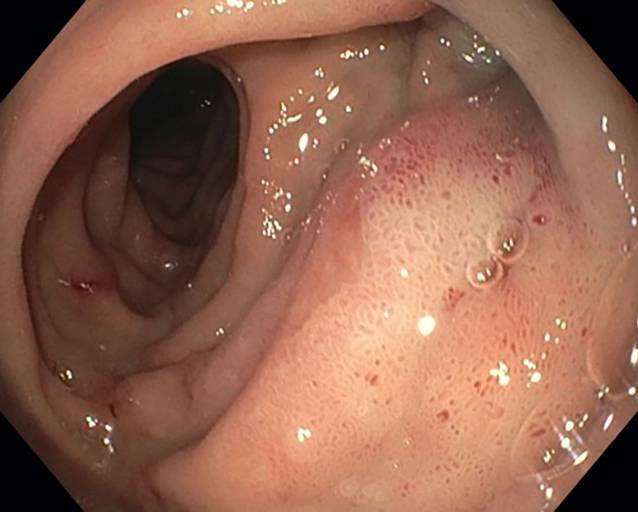
Beendigung der Unterdrucktherapie nach 6 Tagen am 7. postoperativen Tag, die Perforationsstelle ist abgeheilt, nur noch geringe oberflächliche Entzündungszeichen sind zu sehen

Die präemptive Unterdrucktherapie wurde nach insgesamt 6 Tagen beendet (Abb. 5).

In der computertomographischen Abschlusskontrolle fand sich keine freie intraabdominelle Luft, kein Anhalt für Abszess oder Sekretverhalt. Der orale Kostaufbau konnte über flüssige und passierte Kost problemlos innerhalb weniger Tage erfolgen.

Zusammenfassung PINT: Einlage am Operationstag, Wechsel am 4. postoperativen Tag (POD), Beendigung am 7. POD. Insgesamt 6 Tage Therapiedauer, enterale Ernährung über Ernährungssonde.

**Abb. 6:**
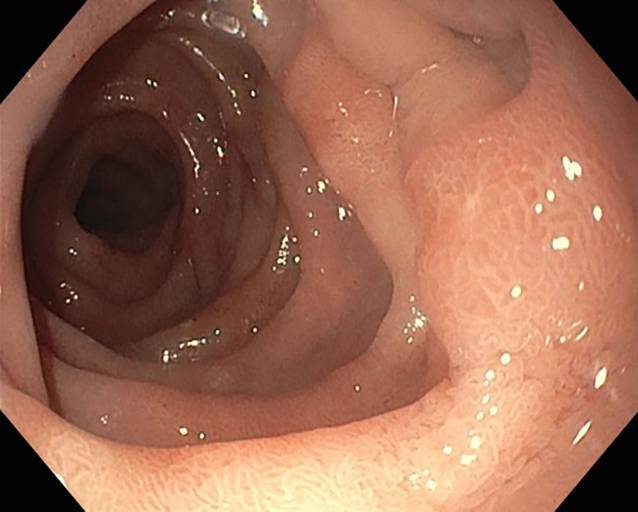
Perforationsdefekt 5 Wochen nach Ereignis mit kompletter Abheilung, es zeigt sich keine Stenose

In einer weiteren Kontrollgastroskopie 5 Wochen nach dem Ereignis stellte sich der duodenale Perforationsdefekt als vollständig abgeheilt dar (Abb. 6).

## Diskussion

Anhand eines Fallbeispiels einer iatrogenen Duodenalperforation zeigen wir, wie die PINT im Duodenum in Ergänzung zur operativen Therapie präemptiv angewendet werden kann. Wir demonstrieren die Technik mit einer offenporigen Foliendrainagesonde, welche eine integrierte Ernährungssonde beinhaltet [8]. Die Abheilung des übernähten Duodenaldefektes war problemlos. Komplikationen der Therapie haben wir nicht beobachtet.

Der Defektverschluss im Duodenum erfolgte bei unserem Patienten operativ. Auch mit endoskopischen Verfahren, beispielsweise mittels OTSC [2], lassen sich Perforationsdefekte verschließen. Die endoskopische Therapie kann immer dann in Erwägungen gezogen werden, wenn die Verletzung von luminalseitig erfolgte. In solchen Fällen kann auch von luminalseitig ein Verschluss mit einer endoskopischen Methode indiziert sein. Anders ist es, wenn, wie bei unserem Patienten, eine Perforation durch eine Punktion von der extraluminalen Seite des Duodenums herbeigeführt wurde. Bei einem solchen Verletzungsmuster muss operativ geklärt werden, ob nicht noch andere relevante extraluminale Strukturen verletzt wurden oder eine extraluminale Infektion vorliegt. Der alleinige endoskopische von luminal herbeigeführte Verschluss wäre möglicherweise nicht ausreichend.

Die präemptive Anwendung der Unterdrucktherapie hat sich aus der therapeutischen ENPT entwickelt. Bei ösophagealen Defekten kann die ENPT heute als Methode der ersten Wahl mit einer hohen Erfolgsrate angesehen werden [9] Intrakavitär und intraluminal werden offenporige Unterdruckdrainagen verwendet, an die mittels einer elektronischen Pumpe ein Unterdruck angelegt werden kann [3]. Zwei kommerzielle Drainagesysteme, die mit offenporigen Polyurethanschäumen ausgerüstet sind, sind zurzeit für den oberen Gastrointestinaltrakt kommerziell erhältlich (Eso-Sponge®; B. Braun SE, Melsungen, Deutschland; Suprasorb® CNP endo Oral und Oral‑N mit Suprasorb® CNP endo Therapieeinheit, Lohmann & Rauscher International GmbH & Co. KG, Rengsdorf, Deutschland). Offenporige Unterdruckdrainagen lassen sich auch mit einer dünnen Drainagefolie (Suprasorb® CNP Drainagefilm, Lohmann & Rauscher International GmbH & Co. KG, Rengsdorf, Deutschland) konstruieren. Dieser von uns entwickelte Drainagetyp ist kleinkalibrig (Durchmesser 4–6 mm), sodass auch eine Platzierung durch kleine Öffnungen und die transnasale Platzierung möglich sind [3, 8]. Die Sonde kann mit einer Ernährungssonde ausgerüstet werden, die dann die simultane Ernährung ermöglicht. Neben dem Einsatz bei Ösophagusdefekten ist das Indikationsspektrum der ENPT mit diesen neuartigen folienbasierten Drainagetypen außerordentlich breit [5, 10].

Die ENPT ist auch zur Behandlung von Duodenaldefekten geeignet [7, 11]. Seit 2010 setzen wir sie im Duodenum ein. Es werden sowohl polyurethanschaumbasierte als auch folienbasierte Drainagen verwendet [7]. Von den beiden chirurgischen Behandlungsprinzipien (Defektverschluss und Sekretdrainage) ist im Duodenum die Drainage der duodenalen Verdauungssekrete in Richtung des Duodenallumens besonders wichtig. Galle lässt sich mit offenporigen Unterdruckdrainagen gut und einfach nach luminal ableiten. Die ENPT ermöglicht die lumenwärts gerichtete, aktive Elimination. Hierdurch wird die enzymatische Kontamination der inneren Wunde unterbunden [7, 8, 11]. Für die PINT wird die intraluminale Variante der ENPT angewendet. Die Drainagen werden mit dem Drainageelement aus Schwamm oder Folie intraluminal in Höhe der Behandlungsregion platziert. Wir wenden die Methode in ausgewählten Fällen an, wenn uns durch die Elimination der Verdauungssekrete in der frühpostoperativen Heilungsphase eine günstige Beeinflussung der inneren Wundheilung erforderlich erscheint. In vielen Fällen konnten wir diese Beobachtung an verschiedenen Anastomosen machen, sodass wir seit 2017 das Verfahren standardmäßig als Sicherheitskonzept bei Ösophagusresektionen in unserem Hause durchführen. Auch hier setzen wir den vorgestellten folienbasierten doppellumigen Drainagetyp mit integrierter Ernährungssonde ein [6].

Eine Ableitung der gastralen und duodenalen Sekrete mit passiven Ableitsonden führt nicht zu einer vollständigen Entleerung des Magens oder Duodenums. Diese Beobachtung kann jeder Endoskopiker, der solche Patienten untersucht, aus eigener Erfahrung bestätigen. Bei der aktiven Drainage mittels offenporiger Drainagen aus Polyurethanschaum oder Drainagefolie hingegen kann eine vollständige Elimination der Sekrete gelingen.

Wir verwendeten eine kleinkalibrige offenporige Foliendrainage. Diese dünne Drainage hat den Vorteil, dass sie sich einfach transnasal wie eine herkömmliche gastroduodenale Ableitsonde einführen lässt. Auch die Passage entlang des Pylorus in das Duodenallumen bereitet keine Schwierigkeiten. Aus zahlreichen Anwendungen mit Schaumdrainagen wissen wir, dass die Passage eines voluminösen Polyurethanschaumkörpers über den Pylorus gelegentlich auch eine Herausforderung darstellen kann.

Auch nach komplexen endoskopischen Interventionen im Duodenum und Ösophagus wird die ENPT mit schaumbasierten Drainagen bereits präemptiv eingesetzt. Hochberger et al. schlugen den präemptiven Einsatz der ENPT zur Verhinderung einer sekundären Perforation nach endoskopischer Resektion großer Duodenalpolypen vor [4]. Aktuell berichteten Blasberg et al. über den prophylaktischen Einsatz im Ösophagus bei ausgedehnter submukosaler Dissektion des oberflächlichen Ösophaguskarzinoms [1].

Alle offenporigen Drainagen können verstopfen. Sie verlieren dann an Sogwirkung und werden ineffektiv. Aus diesem Grund ist ein regelmäßiger Drainagewechsel mit innerer Wundkontrolle notwendig. Unsere endoskopischen Untersuchungsintervalle waren sehr kurz, dieses führte zu einer erhöhten Anzahl an Endoskopien, die ein Risiko für den Patienten darstellen können. Ein Untersuchungsabstand von 3 bis4 Tagen 2‑mal wöchentlich hat sich nach unserer Erfahrung bei der ENPT bewährt. In unserem Fall traten hierdurch keine Komplikationen auf.

Ein Nachteil könnte in der Tatsache gesehen werden, dass ein oraler Kostaufbau erst nach Entfernung der Unterdruckdrainage erfolgt. Allerdings konnten wir zeigen, dass mit einer doppellumigen Drainage eine sofortige enterale Ernährung über die Ernährungssonde simultan zur Unterdrucktherapie unmittelbar postoperativ stattfinden kann.

## Konklusion

Wir berichten über den präemptiven Einsatz der ENPT als PINT bei einem operativen Verschluss einer iatrogenen Duodenalperforation. Die neue Methode hat zum Ziel, die Wundheilung der duodenalen Wunde in der ersten vulnerablen Phase zu unterstützen. Hierzu werden mit einer aktiven Unterdruckdrainage die duodenalen Verdauungssekrete postoperativ eliminiert. Die verwendete kleinkalibrige offenporige doppellumige Foliendrainage ist mit einer Ernährungssonde ausgerüstet. Simultan zur präemptiven ENPT kann die enterale Ernährung erfolgen.

Weitere Studien sind erforderlich, um den Stellenwert der PINT zu evaluieren.

## Abkürzungen

DE: Drainageelement
dOFD: Doppellumige offenporige Foliendrainage
ENPT: Endoscopic negative pressure therapy
EVT: Endoscopic vacuum therapy
iT: Intestinal tube
LWK: Lendenwirbelkörper
NKBS: Nierenbeckenkelchsystem
P: Perforationsdefekt
PARD: Präemptive aktive Refluxdrainage
PINT: Preemptive intraluminal negative pressure therapy
POD: Postoperative day
